# Methylated DNA and microRNA in Body Fluids as Biomarkers for Cancer Detection

**DOI:** 10.3390/ijms140510307

**Published:** 2013-05-16

**Authors:** Yanning Ma, Xian Wang, Hongchuan Jin

**Affiliations:** Laboratory of Cancer Biology, Department of Medical Oncology, Institute of Clinical Science, Sir Runrun Shaw Hospital, Medical School of Zhejiang University, Hangzhou 310029, China; E-Mails: mayanning0604@163.com (Y.M.); wangx118@yahoo.com (X.W.)

**Keywords:** cancer, biomarker, DNA methylation, miRNAs

## Abstract

Epigenetic alterations including DNA methylation and microRNAs (miRNAs) play important roles in the initiation and progression of human cancers. As the extensively studied epigenetic changes in tumors, DNA methylation and miRNAs are the most potential epigenetic biomarkers for cancer diagnosis. After the identification of circulating cell-free nuclear acids, increasing evidence demonstrated great potential of cell-free epigenetic biomarkers in the blood or other body fluids for cancer detection.

## 1. Introduction

Scientists have been engaged in dissecting the mechanism of carcinogenesis for decades and cancer was believed to be a genetic disease. Recently, epigenetics has attracted considerable attention and cancer was recognized as a disease of gene regulation. Although Conrad Waddington coined the word “epigenetics” (literally “over” or “upon” genetics) in the early 1940s to describe the discipline in biology that studies “the interactions of genes with their environment that bring the phenotype into being”, it was currently referred more specifically to heritable changes in gene regulation that are not attributed to changes in DNA sequence [1].

Epigenetics is essential to maintain normal physiological processes to coordinate cell division and tissue-specifically differentiation in eukaryotic organisms. Epigenetic changes involve every aspect of gene regulation in response to environmental epimutagens such as the accessibility of chromosomal DNA to transcription factors and the translation efficiency of mRNA into proteins [2]. Epigenetic deregulations lead to a wide variety of pathological states such as cancers [3]. An explosion of data indicating epigenetic events associated with virtually every step of tumor development and progression, has led to the realization that epigenetic alterations cooperative with genetic abnormalities play important roles in the initiation and progression of human cancers [4,5]. Epigenetic alterations are believed to occur early in tumor development and may precede genetic changes, thus providing the possibilities of early diagnosis even prevention with the development of epigenetic biomarkers [6]. The emergence of advanced technologies to detect genome-wide epigenetic changes holds promise to advance our capacity to develop such biomarkers for detecting cancers at early stage [7].

DNA methylation, the addition of a methyl group to the cytosine pyrimidine ring, is important to maintain genome structure and regulate gene expression. Non-coding RNAs such as miRNAs could regulate gene expression by controlling mRNA stability and translation in addition to gene transcription. Recently, methylated DNA and miRNAs were found to be detected readily in the tissues even bloods, indicating that these epigenetic biomarkers could be the next generation of biomarkers for cancer detection. In this review, we will briefly overview recent advances in epigenetics and mainly discuss the development of DNA methylation and miRNAs as biomarkers for cancer detection.

## 2. Methylated DNA as Biomarker

### 2.1. Overview of DNA Methylation

The most extensively studied epigenetic modification in humans is DNA methylation. DNA methylation is a covalent modification that primarily occurs at Carbon-5 position of cytosine within CpG dinucleotides in mammals. It is mediated by a class of enzymes known as the DNMTs. Currently, several members of the DNMT family have been identified in mammals: DNMT1, DNMT1b, DNMT1o, DNMT1p, DNMT2, DNMT3a, DNMT3b and DNMT3L. DNMT 3A and DNMT3B are *de novo* enzymes and DNMT3L is an accessory enzyme for DNMT3a [8] while DNMT1 maintains the existing methylation pattern following DNA replication [9]. CpG dinucleotides scattered in the human genome are concentrated in short CpG-rich DNA regions called CpG islands that locate in approximately 60% of human gene promoters and in regions of large repetitive sequences such as centromeres and retrotransposon elements [9–12]. While DNA methylation in repetitive sequences could be essential to prevent chromosomal instability and maintain chromosomal integrity, the dynamic methylation of CpG islands associates with the activity of gene expression during development and cell differentiation [13–17]. Methylated CpG islands hinder the binding of activating transcriptional factors to DNA sequences [18,19] or recruit inhibitory proteins such as histone deacetylases (HDACs) [20,21], thus leading to the silencing of genes. X chromosome inactivation and genomic imprinting are classical examples of such an epigenetic regulation [9]. During the initiation and progression of human cancers, many important tumor suppressor genes undergo silencing, thus affecting cellular signal pathways pivotal to carcinogenesis [22]. Recently, accumulating evidence suggests that epigenetic deregulation may precede the classical genetic changes such as mutations in tumor suppressors or oncogenes. Therefore, the detection of DNA methylation could reflect the early development of cancers.

### 2.2. Methods to Detect DNA Methylation

Given the landmark changes in human cancers, DNA methylation shows great promise as biomarkers for early cancer detection, prognosis and prediction [17,18]. With the advance of research on epigenomic alterations, the focus of recent studies has switched from methylation of a single locus in specific tumor suppressor promoters to a genome-wide methylation pattern [23–25]. Basically, all techniques developed for the detection of DNA methylation can be grouped into three classes according to the theoretical principles: sodium bisulfite conversion-dependent methods, restriction enzyme-dependent methods and affinity enrichment-dependent methods. As there is a wide array of technologies developed for DNA methylation detection, we only elucidate the representative ones as examples here.

The majority of methods are developed on the basis that sodium bisulfite deaminates unmethylated cytosines to uracil but leaves methylated cytosines unconverted [26]. After bisulfite conversion, there are a number of techniques available to determine CpG island methylation such as pyrosequencing, quantitative methylation-specific polymerase chain reaction (qMSP), methylation-sensitive single nucleotide primer extension (MS-SNuPE), bisulfate methylation profiling (BiMP), methylation-specific quantum dot fluorescence resonance energy transfer (MS-qFRET) and whole-genome shotgun bisulfite sequencing (WGSBS). Of the various techniques available, MSP is the most frequently used method to detect DNA methylation and qMSP seems to be superior in the detection of minute amounts of methylated DNA [27–30]. In this assay, primers are designed to overlap the CpG site of interest in the DNA template after sodium bisulfate treatment. It allows determining methylation states of particular sites sensitively and cost-effectively but with high false-positive rate since a small subset of the DNA copies have a substantially lower conversion rate [31]. In contrast to MSP that can only reflect the methylation status of single or few CpG sites within the primer sequence, Bisulfite Genomic Sequencing (BGS) can sequence multiple CpG sites within the amplicons when primers are designed to anneal with DNA sequence lacking CpG sites. When sodium bisulfate treatment is performed under appropriate conditions, the expected conversion efficiency of unmethylated cytosines can be up to 99% [32]. However, many factors such as contamination of proteins, strand separation efficiency and DNA quality contribute to the unsatisfactory conversion efficiency, thus limiting the wide application of bisulfite-conversion dependent methods clinically [31]. MS-qFRET combines the high specificity of MSP and the high sensitivity of the quantum dot FRET (QD-FRET) technology, showing the advantage of its potential application for high-throughput screening in multiplexing reactions [33]. An alternative approach for bisulfite treated DNA is matrix-assisted laser desorption/ionization time of flight (MALDI-TOF) mass spectrometry [34,35]. Although it is not a genome-wide scale technology, it can be reliably applied to pooled DNA samples to obtain group averages and can provide accurate results of multiple CpG dinucleotides for hundreds of gene loci.

Another class of techniques, restriction enzyme-dependent methods, utilizes the different ability of methylation sensitive/insensitive restriction enzymes to recognize and cleave given DNA sequences. The disadvantage of these methods is only a particular pattern of CpG sites can be analyzed. Many advanced techniques have been developed that couple enzymatic digestion to array-based hybridization. Differential methylation hybridization (DMH), MCA with microarray hybridization (MCAM), HpaII tiny fragment enrichment by ligation-mediated PCR (HELP) are examples of this class. Another method, methylation-specific multiplex ligation-dependent probe amplification (MS-MLPA), which intergrats the MLPA technique with methylated specific restriction enzymes, has proved to be a semiquantitative and convenient technique for evaluating the methylation status of multiple sequences simultaneously in tissue samples [36]. In a modified method termed Combined Bisulfite Restriction Analysis (COBRA), these restriction enzymes are also used to combine with bisulfate conversion to improve the analytical sensitivity and specificity.

In addition, methylated DNA can be enriched by antibodies specific to methylated cytosine or methyl-binding proteins. These approaches include methylated DNA immunoprecipitation (MeDIP) and methylated CpG island recovery assay (MIRA) [37]. Depending on the downstream platform used, enriched methylated DNA could be subject to whole-genome analysis by array-based hybridization or the next generation sequencing as well as gene-specific determination by PCR.

### 2.3. Detection of DNA Methylation in the Blood and Other Body Fluids

DNAs used for methylation analysis are usually extracted from tissues, thus limiting the clinical utility for early cancer diagnosis. In contrast, analysis of methylated DNA in the blood or other body fluids could reflect tumor burden in a non-invasive manner so as to be useful for cancer screening. Despite the first elucidation of cell-free nucleic acids (cf-NAs) in human bloods early in 1948 [38], it is not until 1990s that research on circulating cf-NAs starts to be prosperous. Moreover, epigenetic biomarkers were also detectable in other body fluids such as nipple aspirate [39,40], urine [41,42], sputum [43] and bronchoalveolar lavage [44].

The first analysis of plasma/sera DNA methylation was conducted in breast cancer [45]. After then, researches on plasma/serum and other body fluids have never stopped and demonstrated the potential of DNA methylation as markers for clinical application. Some important results in recent years are summarized in Tables 1 and 2. Many efforts have been dedicated to the discovery of novel methylated DNA for cancer detection. The methylation analysis of several genes rather than a single gene improves the clinical efficacy. For example, methylation of six genes including CYCD2, HIC1, PAX 5, RASSF1A, RB1 and SRBC can differentiate colorectal cancer patients from controls with sensitivity as 84% and specificity as 68% [46]. Similarly, methylation analysis of a gene panel containing APC, BIN1, BRCA1, CST6, GSTP1, P16, P21 and TIMP3 was developed to detect breast cancer with a sensitivity and specificity more than 90% [47]. Recent studies also compared or combined traditional clinical cancer markers with potential DNA methylation biomarkers. A genome-wide scale study with plasma and serum samples from 107 colorectal cancer patients and 98 individuals without colorectal cancer revealed that analysis of methylated THBD and C9orf50 outperformed carcinoembryonic antigen (CEA) measurement for early colorectal cancer detection [48]. Moreover, simultaneous measurements of both DNA methylation and carcinoembryonic antigen (CEA) resulted in increased sensitivity and specificity, even when either marker alone had low sensitivity [49].

During clinical validation, parameters such as AUC (area under ROC curve) are common indicators used for evaluation of efficiency. Moreover, the sample size used in different studies affects the significance of the results. However, most of the proposed biomarkers lack convincing ROC analysis mainly due to limited number of cases enrolled in the study. In addition, most studies have only included a small number of healthy control subjects so that the normal patterns of DNA methylation are only poorly characterized. Therefore, most of methylated DNA biomarkers need to be validated by large-scale clinical trials, ideally prospectively, to finalize the convincing specificity and sensitivity. Interestingly, recent studies reported some methylation markers could be detected as positive even in patients with benign diseases [93] or heavy smokers [94]. These findings could account for the relatively unsatisfactory specificity (<90%) of the potential biomarkers [52,57]. More fundamental studies are warranted to design further large-scale clinical trials for biomarker validations.

The first commercial DNA methylation test for the diagnosis of early colorectal cancer (CRC) and endometriosis is the detection of SEPT9 methylation. In a prospective trial with over 7900 average-risk screening guideline-eligible asymptomatic subjects, the first generation of SEPT9 test detected up to 48.2% of the cancer cases with the specificity as 91.5% [59]. However, the low sensitivity (11.2%) for advanced adenomas hinders its clinical utility for cancer screening [59]. Other biomarkers being licensed includes methylated SHOX2 for lung cancer (Epi, proLung, Epigenomics AG) and methylated PITX2 for prostate cancer.

### 2.4. Standardization of Methylation Analysis

As no methods mentioned above are absolutely superior to others considering important assay parameters including high analytical sensitivity and specificity, accuracy, robustness, reproducibility, low risk of sample contamination, studies show different results with various analysis methods. The absence of standard methods affects the credibility of DNA methyaltion as valuable biomarkers for cancer detection. For example, GSTP1 methylation ranges from 21.4% to 73% in urine [41,95,96] and from 36.2% to 72% [95,97] in plasma even with the same technique. The most important variant could be the different conditions in which urine or plasma/serum specimens are collected. Another key problem is the efficiency of DNA extraction and quantification of DNA after the standardization of sampling procedures such as collection and storage. Therefore, DNA methylation detection must be standardized to warrant the efficient development of DNA methylation as biomarkers for cancer [98]. Guidelines including universal individual laboratory protocols should be encouraged for the standardization of methylated DNA analysis.

## 3. MiRNAs as Biomarkers

### 3.1. Overview of miRNAs

MiRNAs are a class of small non-coding RNAs sequences of about 19–24 nucleotides that regulate targeted mRNAs post-transcriptionally to control gene expressions [99]. Precursor miRNAs with hairpin structures are generated from primary transcripts via processing of RNase II Drosha, exported from the nucleus to the cytoplasm in an Exportin-5-dependent manner. Eventually, they are cleaved by Dicer ribonucleases to form the functional mature miRNAs. Mature miRNAs are essential for silencing of gene expression by forming RNA-induced silencing complexes (RISC) to inhibit translation or promote mRNA degradation depending on the degree of their homology to the target sequences [100]. The first miRNAs, lin-4, was discovered in 1993 [101] and up to now, there are 2042 mature human miRNA sequences listed in the miRNA registry (Sanger miRBase, release19; Manchester, UK, 2012).

miRNAs have crucial functions in controlling the expressions of genes involved in virtually all biologic processes such as differentiation, proliferation, cell death, cell-cycle control, metabolism, haematopoiesis and aging [102–105]. Due to genetic and epigenetic changes including deletions or amplification of miRNA genes, epigenetic silencing or inhibition of processing, altered miRNA expression has been reported in various cancers [106,107]. For example, a five-miRNA signature for the prediction of treatment outcome of NSCLC was found in a cohort of 112 samples [108]. In another large sample retrospective analysis, five miRNAs were proved to have prognostic value for patients with nasopharyngeal carcinoma [109]. Recently, a mouse model has been used to identify serum microRNAs (miRNAs) as non-invasive biomarkers for diffuse-type gastric cancer early diagnosis [110]. While most miRNAs are generally downregulated in cancers, a few miRNAs, referred to as oncomiRNAs show elevated expression levels. This phenomenon indicated the potential role of these miRNAs as markers for early detection of cancer occurrence or recurrence in addition to the prediction of prognosis or response to various treatments [111].

### 3.2. Methods to Detect miRNAs

The main methods to detect miRNAs include quantitative RT-PCR (qRT-PCR), deep sequencing, microarray, *in situ* hybridization (ISH), enzymatic luminescence miRNA assay. Currently, qRT-PCR is the most commonly used for miRNA detection which can quantify the miRNAome from minute quantities of individual patient material [112]. Prior to the real time quantitative PCR, miRNAs are reversely transcribed to cDNA using a common RT primer or a pool of stem-loop RT primers specific for each miRNA [113].

A consensus protocol for analyzing miRNAs using qRT-PCR is emerging [114]. As miRNAs are single stranded, techniques that amplify these molecules usually use one unique and one universal primer, which provides less specificity than methods that use two unique primers [115]. The other problem is the normalization of miRNA expression. An ideal solution would be the identification of appropriate internal reference miRNA gene. However, there is currently no consensus on suitable small RNA reference genes [114]. MiR-16 or the small nucleolar RNA RNU62 and SNORD43 are frequently used as reference genes, but recent studies suggested that miR-16 is highly expressed in erythrocytes and its level in the blood can be affected significantly by the hemolysis [55–59]. Besides, RNU62 is less representative as it is not synthesized by the same polymerases that synthesize precursor miRNAs. An alternative normalization method is to establish mean expression levels of all tested miRNAs to reduce the technical variation in the miRNA isolation [116]. Further researches are needed for the uniformed standard to allow better comparisons and validations of miRNA biomarkers in the blood.

Isolation of miRNAs from serum and plasma is relatively straightforward. Exosome isolation can improve miRNA extraction from the circulation as the majority of miRNAs detectable in serum are contained in exosomes [117]. In addition, as some miRNAs are highly expressed in blood cells, the level of plasma miRNA biomarkers can be altered significantly by the various extents of hemolysis [118]. Detecting levels of free hemoglobin and certain miRNAs such as miR-15b and miR-16 may be necessary to determine whether a blood sample is suitable for further miRNA quantitation [119,120]. The standardization of sample processing and normalization of miRNAs analysis methodology is one of the most urgent requirements for preclinical screening and validation so as to facilitate the development of miRNA as biomarkers for clinical application.

### 3.3. Detection of miRNA in Bloods and Other Body Fluids

Specimens of solid cancer for miRNA detection are obtained either by biopsy or surgery. After the identification of circulating nuclear acids, researchers tried to detect circulating miRNAs in body fluids such as serum and plasma. Although the underlying mechanisms remain poorly understood, miRNAs in plasma or sera seem to be in a remarkably stable form that is resistant to RNase digestion even under harsh conditions including boiling, low/high pH, extended storage time, and freeze-thaw cycles [121]. Hence, the levels of miRNAs in serum are stable, reproducible, and consistent among individuals of the same species even for several years [122]. In 2008, Lawrie, C.H. *et al.* described the presence of miRNAs in serum of cancer patients for the first time and found the association of miR21 with relapse-free survival of patients with diffuse large B cell lymphoma [123]. Blood-based miRNA expression profiles have since been shown to be potential biomarkers in cancer diagnosis and prognosis (Table 3). For example, serum level of miRNA-141 level was increased in the patients with prostate cancer and was able to detect prostate cancer with 100% specificity and 60% sensitivity [124]. In addition, miR-141 level in the blood was demonstrated to have a high correlation with other classical biomarkers particularly PSA [125].

MiRNAs have also been detected in other body fluids such as urine, tears, breast milk, bronchial lavage as well as pleural, peritoneal, and cerebrospinal fluids [174,175]. For example, increased levels of miRNAs in the urine often indicate the occurrence of urogynaecological cancers mainly bladder cancers and prostate cancers [176]. Similarly, miRNAs such as miR-205 was detectable in the sputum of patients with aerodigestive cancers like lung cancer [177]. In contrast, some miRNAs are downregulated in body fluids of cancer patients although the detailed mechanism remains unknown. For example, the levels of miR-125a and miR-200a were significantly lower in saliva of patients with oral squamous-cell carcinoma (OSCC) [178]. In pleural effusion from patients with lung cancer, the level of miR-198 was also decreased. When combined with the detection of CEA and CYFRA 21-1, miR-198 quantification even improved the sensitivity and specificity for the diagnosis of lung cancer [179].

## 4. Conclusions and Perspectives

In recent decades, the role of epigenetic alterations in carcinogenesis has received greater attention more than ever before. After elucidating the fundamental role of epigenetic changes in human carcinogenesis, considerable efforts have been devoted to the development of epigenetic biomarkers for cancer detection or monitoring and prognosis prediction [7,180]. Presence of cell-free methylated-DNA and miRNAs in blood opened up new perspectives in the development of cancer biomarkers for early cancer detection in a non-invasive manner. Encouraging results have been obtained using advanced techniques with high sensitivity and specificity. Undoubtedly, the diagnostic value of epigenetic molecules in panels or in combination with the conventional clinical biomarkers could be superior to individual markers [46,47,49]. In addition, it is urgent to standardize the methodologies including sample storage and DNA or miRNA extraction to translate the quantitation of circulating epigenetic biomarkers into a clinical routine for cancer diagnosis and prognosis predication [181,182].

## Figures and Tables

**Table 1:** DNA methylation in plasma and serum as cancer markers.

Markers	Source	Sample number	Sensitivity	Specitivity	Technology	Ref.
**Breast cancer**						
ESR1,14–3-3-r	Serum	274	81%	88%	qMSP	[50]
SLC19A3	Plasma	78	90%	85%	qMSP	[51]
GSTP1,RARB, RASSF1, APC	Plasma	169	62%	87%	qMSP	[52]
SOX17	Plasma	139	37%	98%	MSP	[53]
DKK3, ITIH5	Serum	243	40%	93%	MSP	[54]
RASSF1A,DKK3, ITIH5	Serum	243	67%	69%	MSP	[5]
APC, BIN1, BMP6, BRCA1, CST6, ESR-b, GSTP1, P16, P21 and TIMP3	Plasma	126	>90%	>90%	EpiTYPER	[47]
**Colorectal cancer**						
ALX4	Serum	82	83%	70%	qMSP	[55]
CDH4	Peripheral Blood	63	70%	100%	MSP	[56]
NGFR	Plasma	312	51%	84%	qMSP	[57]
SEPT9	Plasma	312	69%	86%	qMSP	[57]
TMEFF2	Plasma	312	65%	69%	qMSP	[57]
RUNX3	Serum	75	68%	89%	MSP	[58]
SEPT9	Plasma	1510	77%	91%	qMSP	[59]
MGMT	plasma	583	39%	96%	MSP	[29]
RARβ2	Plasma	583	24%	100%	MSP	[29]
RASSF2A	Plasma	583	58%	100%	MSP	[29]
Wif-1	plasma	583	74%	98%	MSP	[29]
**Lung cancer(NSCLC)**						
SHOX2	Plasma	411	60%	90%	qMSP	[60]
APC, RASSF1A, CDH13, KLK10 and DLEC1	Plasma	160	83%	70%	MSP	[61]
DLEC1	Plasma	128	36%	98%	MSP	[62]
RARβ2	Plasma	141	28%	48%	MSP	[63]
CDH1	Serum	106	62%	70%	qMSP	[64]
APC, AIM1, CDH1, DCC, MGMT, RASSF1A	Serum	106	84%	57%	qMSP	[64]
CDH13	Plasma	99	33%	83%	MSP	[65]
**Gastric cancer**						
KCNA4, CYP26B1	Serum	92	91%	92%	MSP	[66]
**Hepatocellular carcinoma**						
APC, GSTP1, RASSF1A, SFRP1	Plasma	150	93%	82%	MSRE-qPCR	[67]
TFPI2	Serum	93	46%	72%	MSP	[68]
**Head and neck squamous cell carcinoma**						
CDH1, TIMP3, HIC1, PGP9.5	Serum	251	81	43	qMSP	[69]
**Ovarian cancer**						
BRCA1, HIC1, PAX5, PGR, THBS1	Plasma	66	85%	61%	MethDet test	[70]
RASSF1A, CALCA, EP300	Plasma	60	90%	87%	MethDet test	[71]
**Pancreatic cancer**						
CCND2, PLAU, SOCS1, THBS, VHL	Plasma	60	76%	59%	MethDet test	[72]
NPTX2	Plasma	169	80%	76%	qMSP	[73]
p16	Plasma	83	24%	N/A	MSP	[74]
**Bladder cancer**						
TIMP3, APC, RARB, TIG1, GSTP1, p14, p16, PTGS2, RASSF1A	Serum	148	62%	89%	MSRE-qPCR	[75]
APC, GSTP1, TIG1	Serum	90	80%	93%	qMSP	[76]
**Prostate cancer**						
GSTP1, RASSF1, RARβ2	Serum	123	63%	N/A	MSP	[28]
GSTP1, MDR1	Serum	227	32%	100%	qMSP	[77]
GSTP1, TIG1, PTGS2, RPRM	Serum	210	47%	93%	qMSP	[78]

Sensitivity is defined as the percentage of confirmed cases of disease, in which methylation of a marker is found in serum or plasma; Specificity is defined as the percentage of controls without the disease that are lack of detectable methylation in serum or plasma; N/A: not available; qMSP: quantitative methylated-specific PCR; NSCLC: non-small cell lung cancer; MSRE-qPCR: methylation sensitive restriction enzyme-quantitative PCR.

**Table 2:** DNA methylation in other body fluids as cancer markers.

Markers	Source	Sample number	Sensitivity	Specitivity	Technology	Ref.
**Lung cancer**						
CDKN2A/p16, TERT, WT1, RASSF1	Bronchial washings	248	82%	91%	qMSP	[30]
DAPK, PAX5b, PAX5a, Dal1, GATA5, SULF2, CXCL14	Sputum	130	75%	68%	Nest qMSP	[79]
**Non small cell lung cancer (NSCLC)**						
CDH13	Sputum	190	27%	75%	Nest MSP	[80]
CDKN2A/p16	Sputum	190	40%	73%	Nest MSP	[80]
DAPK	Sputum	190	43%	67%	Nest MSP	[80]
GATA4	Sputum	190	49%	54%	Nest MSP	[80]
IGFBP3	Sputum	190	25%	54%	Nest MSP	[80]
**Head and neck squamous cell carcinoma (HNSCC)**						
MINT31, MGMT, CCNA1, p16	Salivary rinse	391	35%	90%	qMSP	[69]
DAPK, DCC, MINT-31, TIMP-3, p16, MGMT, CCNA1	Salivary	61	54%	N/A	qMSP	[81]
**Prostate cancer**						
GSTP1	Urine	192	81%	94%	qMSP	[82]
RASSF2	Urine	192	59%	63%	qMSP	[82]
HIST1H4K	Urine	192	92%	86%	qMSP	[82]
TFAP2E	Urine	192	100%	18%	qMSP	[82]
GSTP1, RASSF1A, ECDH1, APC, DAPK, MGMT, p14, p16	Urine post massage	95	93%	N/A	MSP	[83]
PCDH17,TCF21	Urine	77	26%	100%	qMSP	[84]
**Colorectal cancer**						
TFPI2	Stool	197	76%–89%	79%–93%	qMSP	[85]
GATA4	Stool	58	71%	93%	MSP	[86]
NDRG4	Stool	58	77%	100%	qMSP	[87]
Vimentin exon-1	Stool	292	46%	90%	MSP	[88]
**Bladder cancer**						
PCDH17,TCF21	Urine	98	60%	100%	qMSP	[84]
GDF15	Urine	71	47%	100%	qMSP	[89]
HSPA2	Urine	71	59%	100%	qMSP	[89]
TMEFF2	Urine	71	63%	100%	qMSP	[89]
VIM	Urine	71	78%	100%	qMSP	[89]
VIM, TMEFF2, GDF15, HSPA2	Urine	71	94%	100%	qMSP	[89]
VAX1, KCNV1, TAL1, PPOX1, CFTR	urine	212	86%	87%	MSP	[90]
ZNF154, POU4F2, HOXA9, EOMES	Urine	174	84%	96%	MSP	[91]
SALL3, CFTR, ABCC6, HPR1, RASSF1A, MT1A, RUNX3, ITGA4, BCL2, ALX4, MYOD1, DRM, CDH13, BMP3B, CCNA1, RPRM, MINT1, BRCA1	urine sediments	168	92%	87%	MSP	[92]
**Renal cell cancer**						
PCDH17, TCF21	Urine	98	32%	100%	qMSP	[84]

Sensitivity is defined as the percentage of confirmed cases of disease, in which methylation of a marker is found in serum or plasma; Specificity is defined as the percentage of controls without the disease that are lack of detectable methylation in serum or plasma; N/A: not available.

**Table 3:** Circulating miRNAs as cancer biomarkers.

Disease	Expression level	Markers	Ref.
**Breast cancer**	Up-regulated	miR-155	[126]
		miR-195	[127]
		miR-10b, miR -34a	[128]
		let7a, miR-195	[129]
		miR-29a, miR-21	[130]
		miR-16, miR-25, miR-222, miR-324–3p	[131]

**Colorectal cancer**	Up-regulated	miR-17–3p,miR-92	[132]
		miR-29a, miR-92a	[133]
		miR-221	[134]
		miR-29a	[135]
		miR-141	[136]

	Down-regulated	miR-34a	[137]

**Gastric cancer**	Up-regulated	miR-17–5p, miR-21, miR-106a, miR-106b	[138]
		miR-106a, miR-17	[139]
		miR-378	[140]
		miR-196a	[141]
		miR-200c	[142]
		miR-17–5p,miR-20a	[143]
		miR-21	[144]

	Down-regulated	let7a	[138]
		miR-195–5p	[145]

**Non-small cell lung carcinoma (NSCLC)**	Up-regulated	miR-25,miR-223	[121]
		miR-17–3p, miR-21,miR-106a, miR-146, miR-155, miR-191, miR-192, miR-203, miR-205, miR-210,miR-212, miR-214	[146]
		miR-1, miR-30d,miR-486, miR-499	[147]
		miR-29c	[148]
		miR-21, miR-205, miR-30d, miR-24	[149]

	Down-regulated	miR-146b, miR-221, let-7a, miR-155, miR-17–5p, miR-27a, miR-106a	[148]

**Pancreatic cancer**	Up-regulated	miR-21, miR-155, miR-196a	[150]
		miR-210	[151]
		miR-200a,miR-200b	[152]
		miR-18a	[153]

**Hepatocellular carcinoma (HCC)**	Up-regulated	miR-500	[154]
		miR-375	[155]
		miR-122	[156]
		miR-15b, miR-21, miR-130b, miR-183	[157]
		let-7a, let-7f, miR-98	[158]
		miR-21, miR-122, miR-223	[159]

**Head and neck squamous cell carcinoma (HNSCC)**	Up-regulated	miR-21, miR-26b	[160]

**Oral squamous cell carcinoma (OSCC)**	Up-regulated	miR-184	[161]
		miR-31,miR-21	[162]
		miR-24	[163]

**Diffuse large B-cell lymphoma (DLBCL)**	Up-regulated	miR-21,miR-155, miR-210	[123]

**Acute myeloid/leukemia (AML), Acute lymphoblastic leukemia (ALL)**	Up-regulated	let-7b, miR-523	[164]

**Multiple myeloma**	Up-regulated	miR-720	[165]
	Down-regulated	miR-1308	

**Prostate cancer**	Up-regulated	miR-141, miR-200b	[124]
		miR-16, miR-34b, miR-92a, miR-92b, miR-103, miR-107, miR-197, miR-328, miR-485–3p, miR-486–5p, miR-574–3p, miR-636, miR-640,miR-766, miR-885–5p	[166]
		miR-221	[167]
		miR-21, miR-221	[168]
		miR-93, miR-106a, miR-874, miR-1207–5p, miR-1274a	[169]

	Down-regulated	miR-145,miR-155	[129]
		miR-24, miR-26b, miR-30c, miR-223	[169]

**Ovarian cancer**	Up-regulated	miR-21, miR-92, miR-93, miR-126, miR-29a	[170]
		miR-21, miR-141, miR-200a, miR-200c, miR-200b, miR-203, miR-205, miR-214	[171]

	Down-regulated	miR -155, miR- 127,miR 99b	[170]

**Serous epithelial ovarian cancer (SEOC)**	Up-regulated	miR200a, miR200b, miR200c	[172]

**Glioblastoma**	Up-regulated	miR-21	[173]
